# Duplication 9p and their implication to phenotype

**DOI:** 10.1186/s12881-014-0142-1

**Published:** 2014-12-20

**Authors:** Roberta Santos Guilherme, Vera Ayres Meloni, Ana Beatriz Alvarez Perez, Ana Luiza Pilla, Marco Antonio Paula de Ramos, Anelisa Gollo Dantas, Sylvia Satomi Takeno, Leslie Domenici Kulikowski, Maria Isabel Melaragno

**Affiliations:** Department of Morphology and Genetics, Universidade Federal de São Paulo, Rua Botucatu 740, CEP 04023-900 São Paulo, Brazil; Department of Pathology, Laboratório de Citogenômica, Universidade de São Paulo, Avenida Dr. Eneas Carvalho de Aguiar 647, CEP 05403-000 São Paulo, Brazil

**Keywords:** 9p duplication, Trisomy 9p, Centromere, FISH, SNP-array, Karyotype-phenotype correlation

## Abstract

**Background:**

Trisomy 9p is one of the most common partial trisomies found in newborns. We report the clinical features and cytogenomic findings in five patients with different chromosome rearrangements resulting in complete 9p duplication, three of them involving 9p centromere alterations.

**Methods:**

The rearrangements in the patients were characterized by G-banding, SNP-array and fluorescent *in situ* hybridization (FISH) with different probes.

**Results:**

Two patients presented *de novo* dicentric chromosomes: der(9;15)t(9;15)(p11.2;p13) and der(9;21)t(9;21)(p13.1;p13.1). One patient presented two concomitant rearranged chromosomes: a der(12)t(9;12)(q21.13;p13.33) and an psu i(9)(p10) which showed FISH centromeric signal smaller than in the normal chromosome 9. Besides the duplication 9p24.3p13.1, array revealed a 7.3 Mb deletion in 9q13q21.13 in this patient. The break in the psu i(9)(p10) probably occurred in the centromere resulting in a smaller centromere and with part of the 9q translocated to the distal 12p with the deletion 9q occurring during this rearrangement. Two patients, brother and sister, present 9p duplication concomitant to 18p deletion due to an inherited der(18)t(9;18)(p11.2;p11.31)mat.

**Conclusions:**

The patients with trisomy 9p present a well-recognizable phenotype due to facial appearance, although the genotype-phenotype correlation can be difficult due to concomitant partial monosomy of other chromosomes. The chromosome 9 is rich in segmental duplication, especially in pericentromeric region, with high degree of sequence identity to sequences in 15p, 18p and 21p, chromosomes involved in our rearrangements. Thus, we suggest that chromosome 9 is prone to illegitimate recombination, either intrachromosomal or interchromosomal, which predisposes it to rearrangements, frequently involving pericentromeric regions.

## Background

Since the report of a 9p duplication by Rethore *et al*. [1], more than 150 patients have been reported in the literature. Trisomy 9p, together with trisomy 21, 18 and 13 are the most common trisomies found in newborns. A possible explanation might be that 9p chromosome is relatively poor in genes and therefore more compatible with survival [2]. In general, there is a remarkable consistency in the phenotype, especially in facial and digital anomalies, despite of variations in the size of the duplication [3,4]. Other features include short stature, microcephaly/brachycephaly, downslanting palpebral fissures, deep set eyes, hypertelorism, short wide neck, globular/prominent nose, short philtrum, downturned corners of the mouth, low set ears, development delay, anomalies of hands and toes and variable degree of intellectual disability [3,5-7]. Trisomy for 9pter → p11 is associated with typical craniofacial features, while trisomy for 9pter → q11-13 shows skeletal and cardiac defects in addition to the craniofacial features [8]. The degree of clinical severity is related to the extension of the 9p duplicated segment [5] but phenotype-genotype correlation studies suggested that the minimal critical region for the classical trisomy 9p is located in 9p22 → p24 [7,9] while Christ *et al*. [10] proposed a shorter region 9p22.1 → p23.

In most cases partial 9p trisomy was the result of parental reciprocal translocations between chromosome 9 and another autosome [11]. Direct 9p duplication was reported only in a few papers [12]. Thus, in the majority of cases, phenotypic heterogeneity occurs due to the variable size of the duplicated segment and the frequent concomitant monosomy or other chromosome segment [11].

We report five patients (two of them siblings) with four different 9p duplications and three of them involving the centromere of chromosome 9. Two patients present pure duplication, one presents 9p duplication associated with 9q deletion and two patients inherited the derivative chromosome from a maternal translocation and both present an 18p deletion associated with the 9p duplication.

### Clinical report

#### Patient 1 (P1)

**Figure 1:**
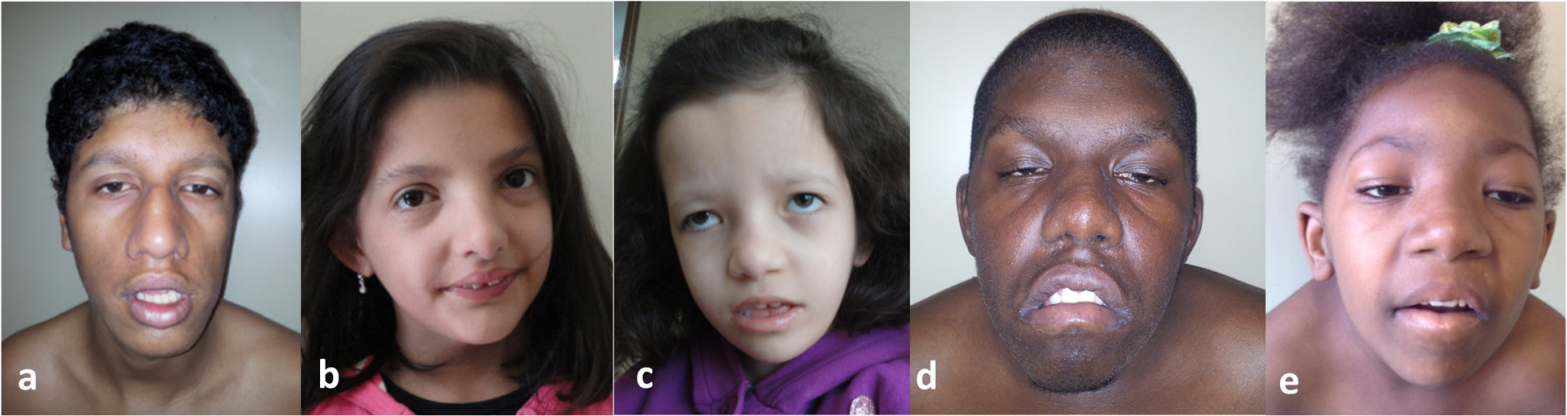
**Patients 1 (a), 2 (b), 3 (c), 4 (d) and 5 (e) at age 14, 5, 7, 17 and 8, respectively.**

Male, unique son from a young non-consanguineous couple and born by cesarean section. At birth, his measurements were: weight of 2,030 g (<3rd centile) and length of 48 cm (15 th centile) and unknown head circumference. At 7 years, his height of 118.5 cm (3rd centile), weight was 23,7 Kg (50 th centile) and head circumference of 47.5 cm (<3rd centile). The clinical evaluation revealed: microcephaly, downslanting palpebral fissures, bilateral microtia, broad and prominent nose, bulbous nasal tip, short philtrum, high palate, scoliosis, bilateral clinodactyly of the 5 th finger, short median phalanges, dystrophic nails in feet and micropenis (Figure 1a). At 17 years and nine months, his height 161.5 of cm (3rd centile); weight was 54,8 Kg, BMI (Body Mass Index) of 21 (15th-50th centile) and head circumference of 51 cm (<3rd centile). He evolved with neuropsychomotor delay and presents with moderated intellectual disability and hipotonia.

### Patient 2 (P2)

Female, second child of a young and non-consanguineous couple. She was born by cesarean section. At birth, her measurements were: weight of 2,400 g (3rd centile), length of 49 cm (50th centile) and unknown head circumference. At 6 years, her weight was 22 kg (75th centile), height of 118 cm (50th centile) and head circumference of 51 cm (50 th centile). The clinical evaluation revealed: brachycephaly, wide forehead, triangular face, low-set ears, broad and prominent nasal bridge, ocular hypertelorism, short philtrum, conchal shelf ears, high and narrow palate, mamarian hypertelorism, bilateral shortening of 2 th finger in hands, bilateral clynodactyly of 5 th fingers in hands, and flat feet (Figure 1b). She evolved with neuropsychomotor delay and recurrent infections.

### Patient 3 (P3)

Female, unique child of a young and non-consanguineous couple. She was born at term by cesarean section after an uneventful pregnancy. At birth, her measurements were: weight 2,620 g (15 th - 50 th centile), length of 49 cm (50 th centile) and head circumference of 32.5 cm (3rd - 15 th centile). At one year, her length was 67 cm (10 th centile), weight was 6,300 g (<3rd centile) and head circumference was 44.5 cm (3rd centile). The clinical evaluation revealed: brachycephaly, wide fontanelles, wide forehead, flattened face, ocular hypertelorism, inversus epicanthus, exotropia, low-set and conchal shelf ears, short nose, long philtrum, kyphosis, brachydactyly, and ungueal hypoplasia. At six years and nine months, her length was 99 cm (<3rd centile), weight was 13,950 g (<3rd centile) and head circumference was 51 cm (50 th centile). The renal ultrasonography showed bilateral renal hypoplasia and the cranial MRI (Magnetic Resonance Imaging) scan revealed mild cranial asymmetry. She evolved with hypotonia, neuropsychomotor delay and seizures (Figure 1c).

### Patient 4 (P4)

Male, first child of a young non-consanguineous couple. His mother is carrier of a balanced translocation and had three pregnancies, one of them an abortion. He was born by cesarean section with birth weight of 3,640 g (15 th - 50 th centile), birth length of 48 cm (15 th centile) and unknown head circumference. At 17 years and three months, the clinical evaluation revealed: height of 163.5 cm (5 th centile); weight of 60 kg, Substitute body mass index by BMI of 22.22 (50 th-75 th centile) and head circumference of 53.5 cm (3rd centile). The clinical evaluation revealed: brachycephaly, coarse face, prominent forehead, ocular hypertelorism, downslanting palpebral fissures, bilateral ptosis, epicanthal folds, prominent nasal bridge, bulbous nasal tip, short philtrum, low set ears, thick lips, downturned corners of the mouth, short and webbed neck, kyphoscoliosis, cryptorchidism, umbilical hernia, hypermobile joints especially in the fingers, braquidactyly, clinodactyly, short toes and flat feet (Figure 1d). The ophthalmic evaluation detected myopia. He presented with hypotonia, neuropsychomotor development and speech delay, and moderate intellectual disability.

### Patient 5 (P5)

Female, sister of patient 4, born by cesarean section with a birth weight of 3,750 g (50 th - 85 th centile); birth length of 49 cm (50 th centile) and unknown head circumference. At six years and seven months, the clinical evaluation revealed: height of 101 cm (<1st centile); weight of 18 kg (15 th centile) and head circumference of 49 cm (3rd centile). The clinical evaluation revealed: proportionate short stature, coarse face, prominent forehead, ocular hypertelorism, exotropia, downslanting palpebral fissures, bilateral ptosis, bilateral epicanthal folds, prominent and large nose, bulbous nasal tip, low set ears, thick lips, large mouth, downturned corners of the mouth, high arched palate, short and webbed neck, kyphoscoliosis, hyperflexible joints especially in the fingers, bilateral clinodactyly of the fifth fingers, short hands and feet, flat feet and dystrophic nails (Figure 1e). The ophthalmic evaluation detected myopia. She presented with hypotonia, neuropsychomotor development and speech delay, and moderate intellectual disability.

## Methods

### Classical and molecular cytogenetic study

This study was approved by the ethics committee of the University Federal of São Paulo (CEP 0389/11). The parent or guardian of the patients signed the consent form for participation in the study and to publish picture. Chromosome analyses were performed on 72-h lymphocyte cultures for all patients (Table 1) and their parents. We performed G-banding and FISH in order to investigate the rearranged chromosomes. FISH was performed with commercial probes for centromere of the rearranged chromosomes (Cytocell®) and BAC (Bacterial Artificial Chromosome) probes RP11-143N16 (9p23) and RP11-373I24 (12p13.33) in the patient 3 as previously described [13].

**Table 1:** **Cytogenomic results for patients with 9p duplication**

**Patient**	**Cytogenomic data**
1	46,XY,der(9;15)t(9;15)(q21.11;p11.2).arr 9p24.3q21.11 (46,586-69,978,010) × 3
2	46,XX,der(9;21)t(9;21)(q21.11;p11.2).arr 9p24.3q21.11(203,861-70,990,047) × 3
3	46,XX,psu i(9)(p10),der(12)t(9;12)(q21.13;p13.33).arr 9p24.3q13(203,861-68,359,990) × 3, 9q13q21.13(68,665,170-76,027,242) × 1
4	46,XY,der(18)t(9;18)(p11.1;p11.31)mat.arr 9p24.3q13(203,861-68,139,972) × 3, 18p11.32p11.31(136,226-6,426,936) × 1
5	46,XX,der(18)t(9;18)(p11.1;p11.31)mat.arr 9p24.3q13(203,861-68,139,972) × 3, 18p11.32p11.31(136,226-6,426,936) × 1

### Molecular studies

DNA was isolated from peripheral blood using a Gentra Puregene kit (Qiagen Sciences Inc., Germantown, MD, USA). High-resolution breakpoint mapping was performed using Cytoscan HD (Affymetrix Inc., Santa Clara, CA, USA) according to the manufacturer’s instructions. The results were analyzed using the Chromosome Analysis Suite (ChAS) software and annotation GRCh37/hg19.

## Results

**Figure 2:**
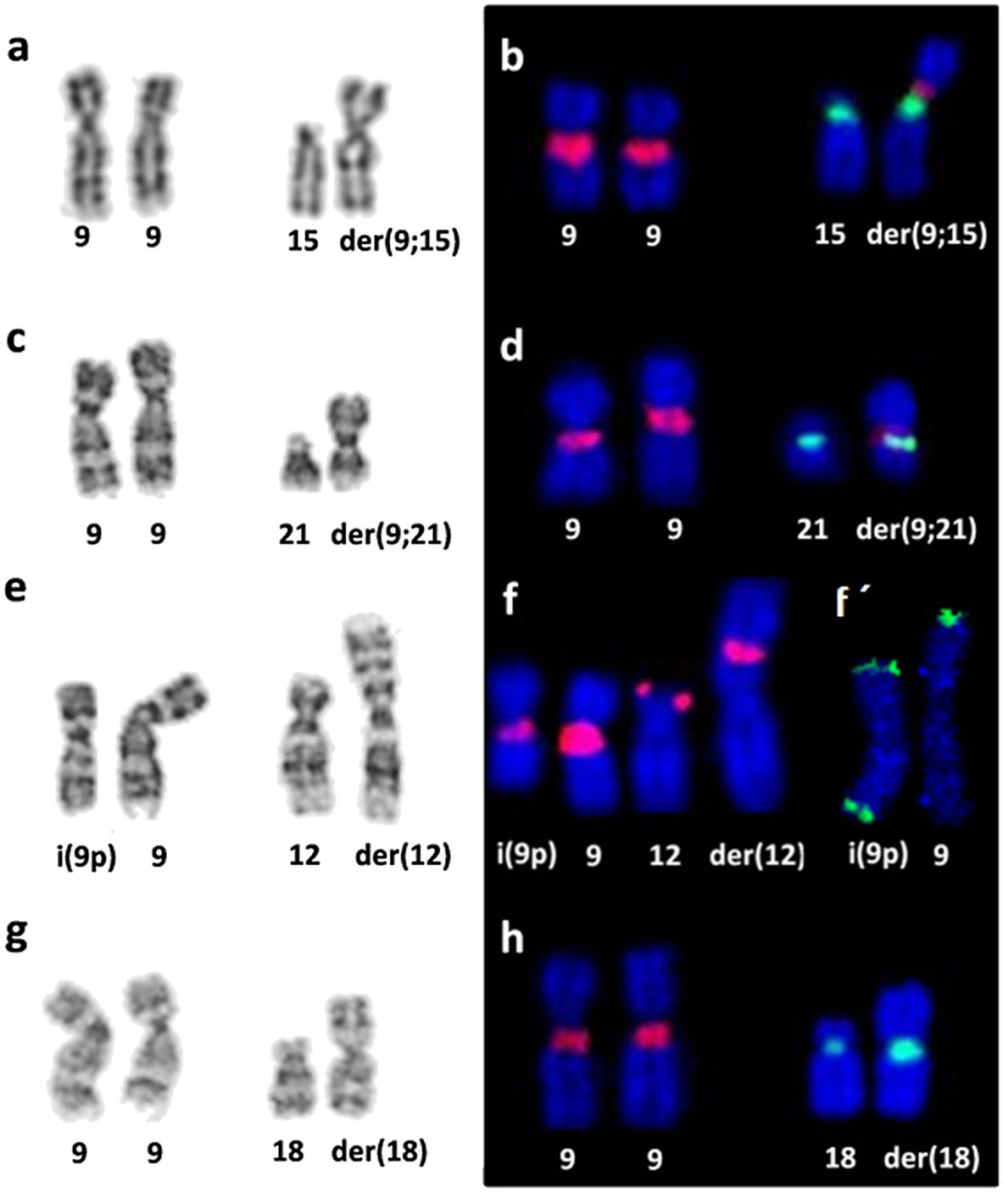
**Partial G-banding karyotype and FISH results in chromosomes involved in the rearrangements in P1 (a-b), P2 (c-d), P3 (e-f) and P4 (g-h).** Two centromeres were identified in P1 **(b)** and P2 **(d)** with centromeric alpha-satellite probes (D9Z1 in red, D15Z1 in green and D21Z1 in green). P3 **(f)** showed a monocentric psu i(9p) with D9Z1 in red, the presence of a subtelomeric region in 12p with the RP11-373I24 BAC probe in red and also (f’) two RP11-143N16 probe green signals in the pseudo-isochromosome 9p. P4 **(h)** showed the der(18) with the centromere for chromosome 18 (D18Z1 in green). P5, who is P4’s sister, present similar karyotype.

Three patients present *de novo* duplications (P1 to P3), while two patients - brother and sister (P4 and P5), present the abnormality inherited from the mother, who has a balanced translocation (Table 1). G-banding and FISH revealed a der(9;15) with centromeres from both chromosomes in P1 (Figure 2a-b), a der(9;21) with also centromeres from both chromosomes in P2 (Figure 2c-d), a pseudo-isochromosome 9p with a small centromere and a der(12)t(9;12) with no deletion in 12p in P3 (Figure 2e, f and f’). P4 and P5 presented a maternal der(18)t(9;18) with a normal chromosome 18 centromere (Figure 2 g-h).

**Figure 3:**
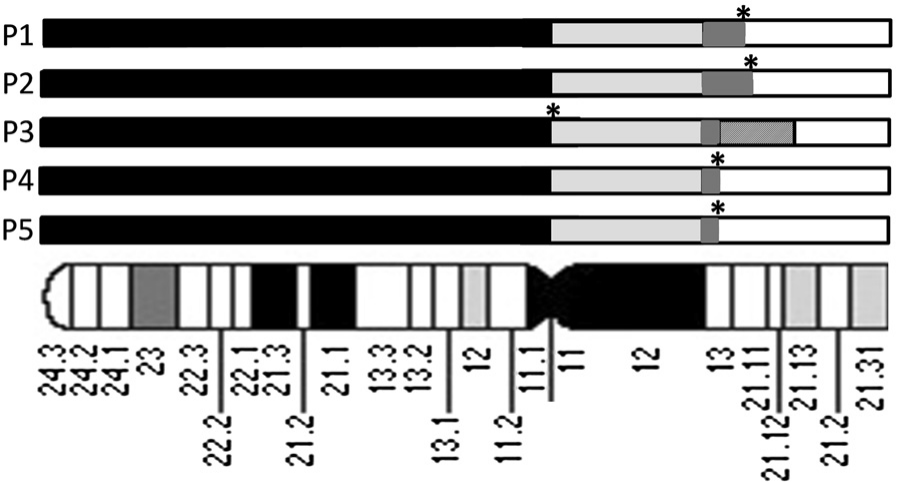
**Partial idiogram of chromosome 9 showing the duplicated regions in black boxes, normal regions in white, heterochromatin regions in light grey boxes, and a deleted region in medium grey box.** The dark grey boxes represent duplicated regions according to array results. The breakpoints (*) were determined by FISH.

Array results (Table 1) revealed complete 9p duplication in all patients. However, patient 3 presented a duplication followed by deletion 9q13 → q21.13, and patients 4 and 5 presented a concomitant 18p deletion due to an unbalanced translocation between chromosomes 9 and 18. Array showed three copies from 9p to proximal 9q in all patients (Figure 3). However, since array presents no probes in the pericentromeric 9p region and its flanking regions are CNVs (Copy Number Variation) rich, we interpreted the breakpoint as being located at 9q21.11 for P1 and P2, considering that both derivative chromosomes are dicentric. For P3, the pseudo-isochromosome 9p presents a fainter FISH signal suggesting the breakpoint within the centromere region. This patient also presents part of the long arm translocated to 12p. For P4 and P5, the breakpoint was mapped at 9p11.1 since FISH does not showed centromeric 9p signal in the derivative chromosome 18 indicating that the break not occurred in the centromere of the chromosome 9. Combining all cytogenomic results, we could characterize the derivative chromosomes as shown in Table 1.

## Discussion

Trisomy 9p is a well-recognizable clinical entity and the facial appearance of patients usually leads to considering the diagnosis [14]. In general, determining the genotype-phenotype correlation is often difficult by the presence of concomitant partial monosomy of other chromosome. We found two patients with pure trisomy 9p (P1 and P2), one with concomitant interstitial deletion 9q (P3) and two with trisomy 9p associated with terminal 18p deletion (P4 and P5).

Table 2 shows the patients reported in the literature in which the breakpoints were determined precisely with high resolution techniques. The clinical characteristics more frequently observed in patients with partial and complete 9p duplication include prominent and large nose, bulbous nasal tip, low set ears, short neck, clinodactyly, neuropsychomotor development delay and speech delay. Some other characteristics are rarely reported, such as strabismus (our P1, P2, P3 and P5), myopia ([15], our P4 and P5), heart disease [9], epilepsy ([16] and our P3), dolichocephaly [17], self-injurious behavior [18] and mild cubitus valgus [15]. The patient described by Bonaglia *et al*. [17] presented average intellectual capability and cognitive functions, dolichocephaly, crowded teeth and high arched palate.

**Table 2:** **Clinical features and chromosome breakpoints in patients with p** **art** **ial and complete 9p duplication defined by FISH or array**

**Features**	**Authors**																	
	**1**	**2**	**3**	**4**	**5**	**6**	**7**	**8**	**9**	**10**	**11**	**12**	**13**	**P1**	**P2**	**P3**	**P4**	**P5**
**9p duplication**	**9p22 → p24**	**9p22 → p24**	**9p22 → p24**	**9p22 → p24**	**9p22 → p24**	**9p24 → 9p21**	**9p24 → p12**	**9p24 → q13**	**9p22.3 → 9p23**	**9p24 → 9q21**	**9p21.3 → 9p13.2**	**9p24.3 → 9p13.1**	**9p24.3 → p13.1**	**9p24.3 → p11.2**	**9p24.3 → p13.1**	9p24.3 → p13.1 *del9q13 → q21.13	9p24.3 → p13 *del18p11.32p11.31	9p24.3 → p13 *del18p11.32p11.31
	**9 y (girl) P1**	**44 y (father) P2**	**9 m (boy)**	**19 m (girl)**	**14 y (boy)**	**6 y (boy)**	**10 m (boy)**	**1 m (girl)**	**6 y (girl)**	**At birth (girl)**	**12 y (girl)**	**15 m**	**13 y (girl)**	**14 y (boy)**	**6 y (girl)**	**6 y (girl)**	**17 y (boy)**	**6y (girl)**
Microcephaly			+	+	+		+	+					+	+	-	-	-	-
Brachycephaly							+	+							+	+	+	
Epicanthal folds			+		+		+						+			+	+	+
Micrognathia					+	+	+								+		-	-
Downslanting palpebral fissures		+	+	+		+		+						+		+	+	+
Prominent/large nose	+	+	+	+		+								+	+	+	+	+
Bulbous nasal tip						+	+	+			+		+	+		+	+	+
Deep set eyes	+			+	+	+	+					+				+	-	-
Hypertelorism				+			+	+							+	+	+	+
Low set ears		+	+	+	+	+		+		-			+		+	+	+	+
Malformed ears				+	+					-					+	+		
Downturned corners of the mouth			+		+		+	+		-			+				+	+
Thin upper lip	+	+				+	+					+					-	-
Short neck		+		+	+	+	+	+			+	+				+	+	+
Fifth finger short			+	-												+		
Nail hypoplasia			-	-	+		+			+				+		+		
Clinodactyly	+	+		-	+	+		+		+	+	+		+	+	+	+	+
Brachydactyly	+	+											+			+		
Neuropsychomotor development delay	+		+	+	+		+	+			+	+	+	+	+	+	+	+
Hypotonia			+	+										+		+	+	+
Growth delay			+		+						+	+		+		+	+	
Small penis					+									+				
Speech delay					+	+					+	+	+			+	+	+

(1-2) Haddad *et al*. 1996 [9]; (3) Fujimoto *et al*. 1998 [7]; (4) Guanciali-Franchi *et al*. 2000 [12]; (5) de Pater *et al*. 2002 [4]; (6) Sanlaville *et al*. 1999 [31]; (7) Tsezou *et al*. 2000 [3]; (8) Teraoka *et al*. 2001 [8]; (9) Bonaglia *et al*. 2002 [17]; (10) Morrissette *et al*. 2003 [30]; (11) Zou *et al*. 2009 [15]; (12) Abu-Amero *et al*. 2010 [16]; (13) Chen *et al*. 2011 [18]; (14-17) Our patients (P1, P2, P3, P4 and P5); (+) present; (-) absent; ( ) not mentioned in the paper * (additional deletion).

Based on the UCSC Genome Browser database (https://genome.ucsc.edu), the duplicated region in our patients contains several annotated genes. *DMRT1* (doublesex- and mab-3-related transcription factor 1) and *DMRT2* (doublesex- and mab-3-related transcription factor 2) are genes related to gonadal development causing hypospadia, abnormal external genitalia, or XY sex reversal, as well as gonadal dysgenesis [19-21]. Only our male patients (P1 and P4) presented gonadal malformation. *FREM1* (FRAS1-related extracellular matrix 1) gene encodes a basement membrane protein that may play a role in craniofacial and renal development. Although none of our patients presented renal abnormality, P1 presented microcephaly and P2, P3 and P4 present brachycephaly. *PSIP1* (PC4 and SFRS1 interacting protein 1), *SIGMAR1* (Sigma non-opioid intracellular receptor 1), *PAX5* (paired box 5) and *CNTNAP3* (contactin associated protein-like 3) genes are involved in the development of central nervous system and are responsible for learning processes, memory and mood alteration. All of our patients presented intellectual disability. Deletion of *FOXD4* (forkhead box D4) gene is associated with speech and language delays [22] as found in eight patients from Table 2. The genes described above cause abnormalities when deleted or mutated. However, in our patients, these genes are present in three copies, probably resulting in overexpression of these genes causing impairment of its function. It is possible that some of the duplicated genes are dosage-sensitive gene and the interaction with other factors, such as regulatory elements (transcription factors and growth factors), may contribute to phenotype and cerebral malformation. In fact, the phenotypic heterogeneity in trisomy 9p can be caused by the variable expression of duplicated genes, which can change the development phenotype [23].

The patient 3 presented a concomitant 9q13-q21.13 deletion which includes genes associated with skeletal muscle and kidney development such as *PGM5* (phosphoglucomutase 5), *PIP5K1B* (phosphatidylinositol-4-phosphate 5-kinase, type I, beta) and *ZFAND5* (zinc finger, AN1-type domain 5) that may be responsible for her hypotonia and kidney alteration. The concomitant 18p deletion in the patients 4 and 5 includes genes associated with muscle cell elastic fibers involved in regulation of contractile activity such as *EMILIN2* (elastin microfibril interfacer 2), *MYOM1* (myomesin 1) and *MYL12B* (myosin, light chain 12B), probably responsible for their hyperlaxity, hiperextensible fingers and hyperflexible joints.

In most patients, the 9p duplicated segment is derived from a parental reciprocal balanced translocation and is accompanied by a concurrent deletion of another chromosome, such as in our patients 3 to 5, who additionally to the 9p duplication, presented a deletion. For P4 and P5, the deletion seems to be more relevant to their clinical features causing short stature, microcephaly, round face, ptosis and downslanting palpebral fissures. Pure *de novo* duplications of 9p - as in our patients 1 and 2 - are rare, and there are only 15 patients reported [16].

**Figure 4:**
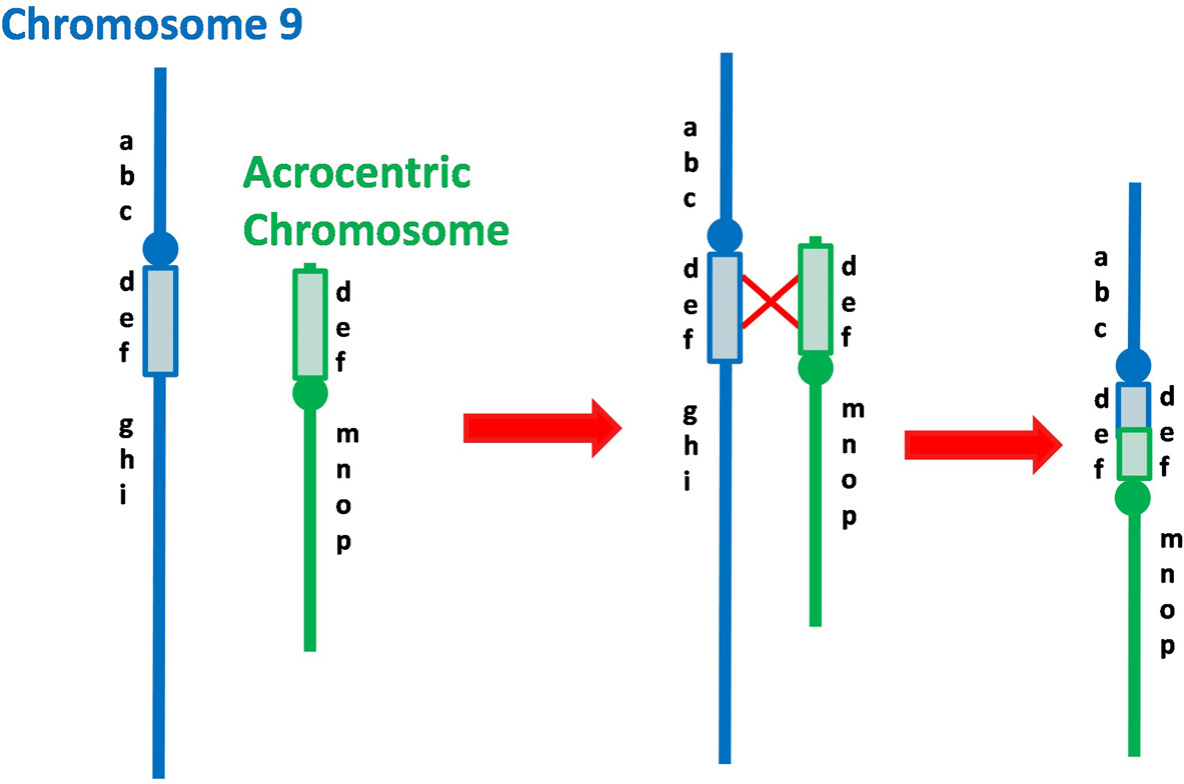
**Schematic presentation showing a proposal mechanism for 9p duplication origin in patients 1 and 2.**

The pericentromeric region of chromosome 9 is especially rich in segmental duplications or low copy repeats (LCR) that predispose it to non-allelic homologous recombination (NAHR) resulting in a high frequency of polymorphic variants located adjacent to the centromere [24], with pericentric inversions (49.4%) being the most frequent alterations [25]. In order to explain our rearranged chromosomes 9, we must consider that at least 5% of the human genome consists of interspersed duplications, either between non-homologous chromosomes (transchromosomal duplications), or restricted to a particular chromosome (chromosome-specific duplications) (Eichler, 2001). In our cases P1 and P2 the unequal recombination between nonhomologous chromosomes would have probably originated 9p duplication that rearranged with other chromosome by NHEJ (nonhomologous end-joining) mechanism possibly due to homology between the breakpoints. Among the interchromosomal segmental duplications, there are some in chromosome 9 with high degree of sequence identity at the nucleotide level DNA to sequences in the pericentromeric region of the short arm of chromosomes 15, 18 and 21, the chromosomes involved in our rearrangements [26-28]. Thus, we suggest that these interchromosomal segmental duplications located in chromosome 9 within the pericentromeric inversion variant, and within the pericentromeric sequences of nonhomologous chromosomes predisposed to illegitimate recombination between them, resulting in the dicentric chromosomes in our P1 and P2 with 9p duplication (Figure 4). These chromosomes may be converted into a stable functional monocentric chromosome by epigenetic centromere inactivation followed by heterochromatinization, which suppresses the revival of centromere activity, as proposed by Sato *et al*. [29] in chromosomes in yeast.

P3 presents a pseudo-isochromosome 9p with a smaller centromere than its homologous, suggesting that the break occurred in the centromere. Probably the deletion 9q (P3) resulted from an error during the translocation with chromosome 12. Patients P4 and P5 inherited a derivative chromosome 18 from their mother. Littooij *et al*. [11] suggested that the relatively high frequency of partial trisomy 9p is not the result of an increased frequency of recombination events due to breakage sensitive regions on 9p, but rather the result of postmeiotic survival of relatively viable zygotes.

FISH was essential to define the rearrangement around the centromeric region of chromosome 9 because arrays do not present probes for the heterochromatin region due to high level of repetitive elements. Moreover, there is a copy number variation around this region making it difficult to interpret the array data. We believe that 9p duplication associated to dicentric chromosomes must be a much more common event than thought since many reported rearranged chromosomes may be insufficiently characterized.

## Conclusion

The patients with trisomy 9p present a well-recognizable phenotype, however the genotype-phenotype correlation can be difficult due to concomitant partial monosomy of other chromosomes, and the possibility of some of the duplicated genes are dosage-sensitive which may influence the phenotype. The use of microarray together with FISH technology was critical for the proper identification and precise molecular characterization of the rearranged chromosomes in order to better define the critical regions, leading to more accurate genotype/phenotype correlations and understanding of the mechanisms involved in these genomic imbalances.

## References

[1] Rethore MO, Larget-Piet L, Abonyi D, Boeswillwald M, Berger R, Carpentier S, Cruveiller J, Dutrillau B, Lafourcade J, Penneau M (1970). 4 cases of trisomy for the short arm of chromosome 9. Individualization of a new morbid entity. Ann Genet.

[2] Venter JC, Adams MD, Myers EW, Li PW, Mural RJ, Sutton GG, Smith HO, Yandell M, Evans CA, Holt RA (2001). The sequence of the human genome. Science.

[3] Tsezou A, Kitsiou S, Galla A, Petersen MB, Karadima G, Syrrou M, Sahlen S, Blennow E (2000). Molecular cytogenetic characterization and origin of two de novo duplication 9p cases. Am J Med Genet.

[4] de Pater JM, Ippel PF, van Dam WM, Loneus WH, Engelen JJ (2002). Characterization of partial trisomy 9p due to insertional translocation by chromosomal (micro)FISH. Clin Genet.

[5] Wilson GN, Raj A, Baker D (1985). The phenotypic and cytogenetic spectrum of partial trisomy 9. Am J Med Genet.

[6] P N: Compendium of birth defects. *Oxford: Blackwell* 1991, In: Buyse ML, ed. :355–356.

[7] Fujimoto A, Lin MS, Schwartz S (1998). Direct duplication of 9p22-p24 in a child with duplication 9p syndrome. Am J Med Genet.

[8] Teraoka M, Narahara K, Yokoyama Y, Ninomiya S, Mizuta S, Une T, Seino Y (2001). Maternal origin of a unique extra chromosome, der(9)(pter-q13::q13-q12:) in a girl with typical trisomy 9p syndrome. Am J Med Genet.

[9] Haddad BR, Lin AE, Wyandt H, Milunsky A (1996). Molecular cytogenetic characterisation of the first familial case of partial 9p duplication (p22p24). J Med Genet.

[10] Christ LA, Crowe CA, Micale MA, Conroy JM, Schwartz S (1999). Chromosome breakage hotspots and delineation of the critical region for the 9p-deletion syndrome. Am J Hum Genet.

[11] Littooij AS, Hochstenbach R, Sinke RJ, van Tintelen P, Giltay JC (2002). Two cases with partial trisomy 9p: molecular cytogenetic characterization and clinical follow-up. Am J Med Genet.

[12] Guanciali Franchi P, Calabrese G, Morizio E, Modestini E, Stuppia L, Mingarelli R, Palka G (2000). FISH analysis in detecting 9p duplication (p22p24). Am J Med Genet.

[13] Kulikowski LD, Bellucco FT, Nogueira SI, Christofolini DM, Smith Mde A, de Mello CB, Brunoni D, Melaragno MI (2008). Pure duplication 1q41-qter: further delineation of trisomy 1q syndromes. Am J Med Genet A.

[14] Concolino D, Cinti R, Moricca M, Andria G, Strisciuglio P (1998). Centric fission of chromosome 9 in a boy with trisomy 9p. Am J Med Genet.

[15] Zou YS, Huang XL, Ito M, Newton S, Milunsky JM (2009). Further delineation of the critical region for the 9p-duplication syndrome. Am J Med Genet A.

[16] Abu-Amero KK, Hellani AM, Salih MA, Seidahmed MZ, Elmalik TS, Zidan G, Bosley TM (2010). A de novo marker chromosome derived from 9p in a patient with 9p partial duplication syndrome and autism features: genotype-phenotype correlation. BMC Med Genetics.

[17] Bonaglia MC, Giorda R, Carrozzo R, Roncoroni ME, Grasso R, Borgatti R, Zuffardi O (2002). 20-Mb duplication of chromosome 9p in a girl with minimal physical findings and normal IQ: narrowing of the 9p duplication critical region to 6 Mb. Am J Med Genet.

[18] Chen CP, Lin SP, Su YN, Chern SR, Tsai FJ, Chen WL, Wang W (2011). Self-injurious behavior associated with trisomy 9p (9p13.1-p24.3). Genet Couns.

[19] Muroya K, Okuyama T, Goishi K, Ogiso Y, Fukuda S, Kameyama J, Sato H, Suzuki Y, Terasaki H, Gomyo H (2000). Sex-determining gene(s) on distal 9p: clinical and molecular studies in six cases. J Clin Endocrinol Metab.

[20] Shan Z, Zabel B, Trautmann U, Hillig U, Ottolenghi C, Wan Y, Haaf T (2000). FISH mapping of the sex-reversal region on human chromosome 9p in two XY females and in primates. Eur J Hum Genet.

[21] Livadas S, Mavrou A, Sofocleous C, van Vliet-Constantinidou C, Dracopoulou M, Dacou-Voutetakis C (2003). Gonadoblastoma in a patient with del(9)(p22) and sex reversal: report of a case and review of the literature. Cancer Genet Cytogenet.

[22] Hauge X, Raca G, Cooper S, May K, Spiro R, Adam M, Martin CL (2008). Detailed characterization of, and clinical correlations in, 10 patients with distal deletions of chromosome 9p. Genet Med.

[23] Bouhjar IB, Hannachi H, Zerelli SM, Labalme A, Gmidene A, Soyah N, Missaoui S, Sanlaville D, Elghezal H, Saad A (2011). Array-CGH study of partial trisomy 9p without mental retardation. Am J Med Genet A.

[24] Willatt LR, Barber JC, Clarkson A, Simonic I, Raymond FL, Docherty Z, Ogilvie CM (2007). Novel deletion variants of 9q13-q21.12 and classical euchromatic variants of 9q12/qh involve deletion, duplication and triplication of large tracts of segmentally duplicated pericentromeric euchromatin. Eur J Hum Genet.

[25] Kosyakova N, Grigorian A, Liehr T, Manvelyan M, Simonyan I, Mkrtchyan H, Aroutiounian R, Polityko AD, Kulpanovich AI, Egorova T (2013). Heteromorphic variants of chromosome 9. Mol Cytogenet.

[26] Wajntal A, Gonzalez CH, Koiffmann CP, de Souza DH (1985). Brief cytogenetic report on maternal translocation t(7;9) (p22:p13): two sibs with duplication 9p and one sib with the balanced translocation. Am J Med Genet.

[27] Di Giacomo MC, Cesarano C, Bukvic N, Manisali E, Guanti G, Susca F (2004). Duplication of 9 p 11.2–p13.1: a benign cytogenetic variant. Prenat Diagn.

[28] Humphray SJ, Oliver K, Hunt AR, Plumb RW, Loveland JE, Howe KL, Andrews TD, Searle S, Hunt SE, Scott CE (2004). DNA sequence and analysis of human chromosome 9. Nature.

[29] Sato H, Masuda F, Takayama Y, Takahashi K, Saitoh S (2012). Epigenetic inactivation and subsequent heterochromatinization of a centromere stabilize dicentric chromosomes. Curr Biol.

[30] Morrissette JJ, Laufer-Cahana A, Medne L, Russell KL, Venditti CP, Kline R, Zackai EH, Spinner NB (2003). Patient with trisomy 9p and a hypoplastic left heart with a tricentric chromosome 9. Am J Med Genet A.

[31] Sanlaville D, Baumann C, Lapierre JM, Romana S, Collot N, Cacheux V, Turleau C, Tachdjian G (1999). De novo inverted duplication 9p21pter involving telomeric repeated sequences. Am J Med Genet.

